# Redetermination of K_4_[Bi_2_Cl_10_]·4H_2_O

**DOI:** 10.1107/S1600536808035435

**Published:** 2008-11-08

**Authors:** Mabrouk Khelifi, Ridha Zouari, Abdelhamid Ben Salah

**Affiliations:** a Faculté des Sciences de Sfax, Laboratoire de Sciences des Matériaux et d’Environnement, BP 115 Sfax 3052, Tunisia

## Abstract

In comparison with the previous refinement of tetra­potassium di-μ-chlorido-bis­[tetra­chloridobismuthate(III)] tetra­hydrate [Volkova, Udovenko, Levin & Shevchenko (1983). *Koord. Khim.* **9**, 356–360], the current redetermination reveals anisotropic displacement parameters for all non-H atoms, localization of the H atoms, and higher precision of lattice parameters and inter­atomic distances. The crystal structure is built up of edge-sharing [Bi_2_Cl_10_]^4−^ double octa­hedra with the bridging Cl atoms situated on a mirror plane, three K^+^ counter-cations (two of which are on mirror planes), and two water mol­ecules that are solely coordinated to the K^+^ cations. These building units are linked into a three-dimensional network structure. Additional O—H⋯Cl hydrogen bonds between the water mol­ecules and the complex anions stabilize this arrangement.

## Related literature

The isotypic Br compound was reported by Laza­rini (1977 ▶). For related structures, see: Belkyal *et al.* (1997 ▶); Benachenhou *et al.* (1986 ▶). For general background, see: Larson (1970 ▶); Prince (1982 ▶); Watkin (1994 ▶).

## Experimental

### 

#### Crystal data

K_4_[Bi_2_Cl_10_]·4H_2_O
*M* *_r_* = 1000.94
Orthorhombic, 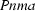

*a* = 8.4310 (1) Å
*b* = 21.8444 (3) Å
*c* = 12.2561 (2) Å
*V* = 2257.21 (6) Å^3^
*Z* = 4
Mo *K*α radiation
μ = 17.49 mm^−1^
*T* = 295 (2) K
0.28 × 0.12 × 0.08 mm

#### Data collection

Bruker APEXII CCD diffractometer
Absorption correction: multi-scan (*SADABS*; Bruker, 2006 ▶) *T*_min_ = 0.067, *T*_max_ = 0.247
26961 measured reflections
4596 independent reflections
2801 reflections with *I* > 3.0σ(*I*)
*R*_int_ = 0.034

#### Refinement

*R*[*F* ^2^ > 2σ(*F* ^2^)] = 0.017
*wR*(*F* ^2^) = 0.017
*S* = 1.08
2801 reflections
114 parameters
All H-atom parameters refined
Δρ_max_ = 1.01 e Å^−3^
Δρ_min_ = −0.82 e Å^−3^

### 

Data collection: *APEX2* (Bruker, 2006 ▶); cell refinement: *APEX2*; data reduction: *APEX2*; program(s) used to solve structure: *SHELXS97* (Sheldrick, 2008 ▶); program(s) used to refine structure: *SHELXL97* (Sheldrick, 2008 ▶) and *CRYSTALS* (Betteridge *et al.*, 2003 ▶); molecular graphics: *DIAMOND* (Brandenburg, 2001 ▶); software used to prepare material for publication: *CRYSTALS* and *publCIF* (Westrip, 2008 ▶).

## Supplementary Material

Crystal structure: contains datablocks I, global. DOI: 10.1107/S1600536808035435/wm2201sup1.cif

Structure factors: contains datablocks I. DOI: 10.1107/S1600536808035435/wm2201Isup2.hkl

Additional supplementary materials:  crystallographic information; 3D view; checkCIF report

## Figures and Tables

**Table 1:** Selected bond lengths (Å)

Bi1—Cl7^i^	2.5954 (8)
Bi1—Cl4	2.6190 (8)
Bi1—Cl2	2.6522 (8)
Bi1—Cl6^i^	2.7205 (7)
Bi1—Cl5^ii^	2.8512 (7)
Bi1—Cl3	2.8724 (7)

Symmetry codes: (i) 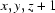; (ii) 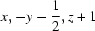.

**Table 2:** Hydrogen-bond geometry (Å, °)

*D*—H⋯*A*	*D*—H	H⋯*A*	*D*⋯*A*	*D*—H⋯*A*
O11—H13⋯Cl7^iii^	0.92 (7)	2.32 (7)	3.234 (4)	169 (7)
O11—H14⋯Cl4^iv^	0.78 (7)	2.56 (8)	3.273 (3)	150 (9)
O12—H8⋯Cl7^v^	0.79 (6)	2.78 (7)	3.497 (4)	152 (7)
O12—H15⋯Cl2^vi^	0.81 (17)	2.81 (8)	3.514 (3)	145 (9)

Symmetry codes: (iii) 
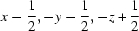
; (iv) 
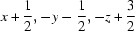
; (v) 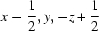; (vi) 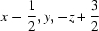.

**Fig. 1.**
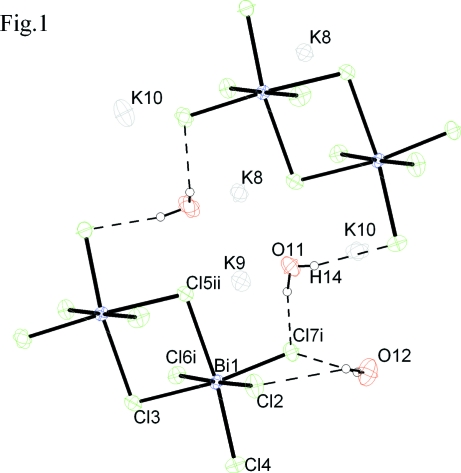
Part of the crystal structure of the title compound with displacement ellipsoids drawn at the 50% probability level. H atoms are shown as spheres of arbitary radius. [Symmetry codes: (i) x, y, z + 1; (ii) -x, -1/2 - y, z + 1.]

**Fig. 2.**
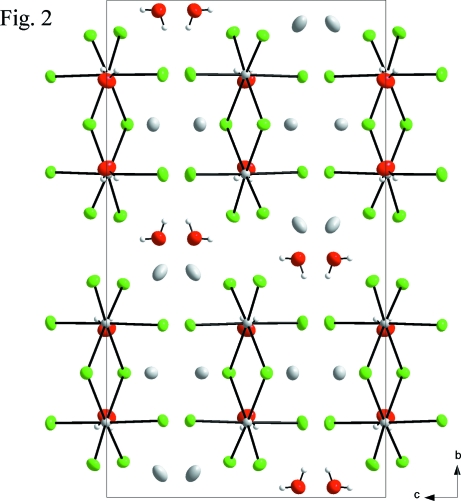
Projection of the K4[Bi2Cl10].4H2O structure along the a axis, showing the pairs of edge-sharing [BiCl6] octahedra.

## supplementary crystallographic information

### Comment

Some bismuth-containing compounds (Belkyal *et al.*, 1997; Benachenhou *et al.*, 1986) exhibit phase transitions and have interesting physical properties which make them the object of an intensive research due to their potential application in catalysis.

Under investigation of a series of these materials, we have selected the K_4_[Bi_2_Cl_10_].4H_2_O compound and redetermined its structure. For the previous crystallographic study on this compound, see: Volkova *et al.* (1983). The isotypic Br compound was reported by Lazarini (1977).

The structure of the title compound can be described by [Bi_2_Cl_10_]^4-^ pairs of octahedra, K^+^ cations and water molecules (Fig. 1), forming a three-dimensional network (Fig. 2). The [Bi_2_Cl_10_]^4-^ anions are formed by pairs of distorted [BiCl_6_] octahedra sharing an edge. The mean Bi—Cl distances range from 2.5954 (8) to 2.8724 (7) Å with the Cl—Bi—Cl angles varying between 80.58 (2) and 94.82 (3)°, likewise showing the distortions of the BiCl_6_ octahedra. Compared to the previous study, the distortion of the [BiCl_6_] octahedra is relatively lower. The structure is additionally stabilized by the presence of hydrogen bonds of the type O—H···Cl between water molecules and the binuclear complex anions. For the polyhedra around the K^+^ cations the coordinations are similar. Whereas two K^+^ cations (K8 and K9) are located at the 4*c* sites (*m* symmetry), the third cation (K10) is on a general position. However, all K^+^ cations are surrounded by two water O atoms and seven Cl atoms, leading to irregular [KO_2_Cl_7_] polyhedra.

### Experimental

(BiO)_2_CO_3_ was dissolved in concentrated hydrochloric acid in order to prepare a BiCl_3_ solution. The latter was then added to an aqueous KCl solution in a molar ratio of 1:2. The resulting solution has been kept under stirring for 1 h and was allowed to stand at room temperature for some days. After this time colourless crystals of the title compound were obtained and isolated from the acid solution by filtration.

### Refinement

The H atom positions were located from difference Fourier maps and were refined freely.

### Crystal data

**Table:**

K_4_[Bi_2_Cl_10_]·4H_2_O	*F*_000_ = 1808
*M**_r_* = 1000.94	*D*_x_ = 2.945 Mg m^−^^3^
Orthorhombic, *P**n**m**a*	Mo *K*α radiation λ = 0.71073 Å
Hall symbol: -P 2ac 2n	Cell parameters from 4596 reflections
*a* = 8.43100 (10) Å	θ = 1.9–34.2º
*b* = 21.8444 (3) Å	µ = 17.49 mm^−^^1^
*c* = 12.2561 (2) Å	*T* = 295 (2) K
*V* = 2257.21 (6) Å^3^	Prism, colourless
*Z* = 4	0.28 × 0.12 × 0.08 mm

### Data collection

**Table:**

Bruker APEXII CCD diffractometer	2801 reflections with *I* > 3.0σ(*I*)
Monochromator: graphite	*R*_int_ = 0.034
*T* = 295(2) K	θ_max_ = 34.2º
ω scans	θ_min_ = 1.9º
Absorption correction: multi-scan(SADABS; Bruker, 2006)	*h* = −10→12
*T*_min_ = 0.067, *T*_max_ = 0.247	*k* = −22→33
26961 measured reflections	*l* = −19→17
4596 independent reflections	

### Refinement

**Table:**

Refinement on *F*	Hydrogen site location: difference Fourier map
Least-squares matrix: full	All H-atom parameters refined
*R*[*F*^2^ > 2σ(*F*^2^)] = 0.017	Method, part 1, Chebychev polynomial, (Watkin, 1994, *P*rince, 1982) [*w*eight] = 1.0/[A_0_*T_0_(x) + A_1_*T_1_(x) ··· + A_n-1_]*T_n-1_(x)] where A_i_ are the Chebychev coefficients listed belo*w* and x = *F* /*F*max Method = Robust Weighting (*P*rince, 1982) W = [*w*eight] * [1-(delta*F*/6*sigma*F*)^2^]^2^ A_i_ are: 0.260 0.753E-01 0.968E-01
*wR*(*F*^2^) = 0.017	(Δ/σ)_max_ = 0.002
*S* = 1.08	Δρ_max_ = 1.01 e Å^−^^3^
2801 reflections	Δρ_min_ = −0.82 e Å^−^^3^
114 parameters	Extinction correction: Larson (1970), Equation 22
Primary atom site location: structure-invariant direct methods	Extinction coefficient: 33.8 (8)
Secondary atom site location: difference Fourier map	

### Fractional atomic coordinates and isotropic or equivalent isotropic displacement parameters (Å^2^)

**Table:**

	*x*	*y*	*z*	*U*_iso_*/*U*_eq_	
Bi1	0.367812 (10)	−0.349919 (4)	0.996881 (8)	0.0204	
Cl2	0.49635 (11)	−0.35057 (4)	0.79938 (7)	0.0394	
Cl3	0.17101 (13)	−0.2500	0.92700 (9)	0.0308	
Cl4	0.15802 (11)	−0.43464 (4)	0.95182 (7)	0.0360	
Cl5	0.56356 (13)	−0.2500	0.06223 (10)	0.0308	
Cl6	0.24213 (9)	−0.34605 (4)	0.20120 (6)	0.0302	
Cl7	0.57912 (10)	−0.42888 (4)	0.05937 (7)	0.0331	
K8	0.44796 (13)	−0.2500	0.34052 (9)	0.0349	
K9	0.28965 (14)	−0.2500	0.66630 (9)	0.0379	
K10	0.02601 (12)	−0.54772 (5)	0.80949 (8)	0.0506	
O11	0.3695 (3)	−0.16188 (16)	0.5001 (3)	0.0504	
O12	0.2459 (5)	−0.47915 (15)	0.6858 (3)	0.0552	
H13	0.278 (9)	−0.140 (3)	0.487 (5)	0.10 (2)*	
H8	0.205 (8)	−0.481 (3)	0.629 (5)	0.09 (2)*	
H14	0.428 (8)	−0.142 (3)	0.535 (5)	0.09 (2)*	
H15	0.223 (5)	−0.445 (8)	0.707 (6)	0.08 (3)*	

### Atomic displacement parameters (Å^2^)

**Table:**

	*U*^11^	*U*^22^	*U*^33^	*U*^12^	*U*^13^	*U*^23^
Bi1	0.02127 (5)	0.01939 (4)	0.02050 (4)	0.00016 (3)	0.00160 (4)	−0.00009 (4)
Cl2	0.0440 (4)	0.0469 (5)	0.0272 (3)	0.0084 (4)	0.0102 (3)	0.0035 (3)
Cl3	0.0218 (5)	0.0365 (6)	0.0342 (5)	0.0000	−0.0043 (4)	0.0000
Cl4	0.0351 (4)	0.0338 (4)	0.0392 (4)	−0.0049 (3)	−0.0057 (3)	−0.0086 (3)
Cl5	0.0228 (5)	0.0305 (5)	0.0390 (5)	0.0000	−0.0037 (4)	0.0000
Cl6	0.0316 (3)	0.0338 (4)	0.0252 (3)	−0.0039 (3)	0.0044 (3)	−0.0028 (3)
Cl7	0.0338 (4)	0.0302 (4)	0.0354 (4)	0.0042 (3)	−0.0028 (3)	0.0025 (3)
K8	0.0329 (5)	0.0334 (5)	0.0383 (5)	0.0000	−0.0061 (4)	0.0000
K9	0.0370 (6)	0.0432 (6)	0.0335 (5)	0.0000	−0.0037 (4)	0.0000
K10	0.0460 (5)	0.0586 (5)	0.0472 (5)	−0.0134 (4)	0.0086 (4)	−0.0190 (4)
O11	0.0291 (13)	0.0507 (16)	0.071 (2)	−0.0020 (11)	−0.0121 (15)	−0.0093 (17)
O12	0.076 (2)	0.0449 (17)	0.0446 (17)	0.0092 (17)	0.0009 (17)	−0.0044 (14)

### Geometric parameters (Å, °)

**Table:**

Bi1—Cl7^i^	2.5954 (8)	Bi1—Cl3	2.8724 (7)
Bi1—Cl4	2.6190 (8)	O11—H13	0.92 (7)
Bi1—Cl2	2.6522 (8)	O11—H14	0.78 (7)
Bi1—Cl6^i^	2.7205 (7)	O12—H8	0.79 (6)
Bi1—Cl5^ii^	2.8512 (7)	O12—H15	0.81 (12)
			
Cl6^i^—Bi1—Cl7^i^	90.94 (3)	Cl6^i^—Bi1—Cl4	87.31 (3)
Cl6^i^—Bi1—Cl5^ii^	86.74 (3)	Cl7^i^—Bi1—Cl4	93.22 (3)
Cl7^i^—Bi1—Cl5^ii^	91.64 (2)	Cl5^ii^—Bi1—Cl4	172.37 (3)
Cl6^i^—Bi1—Cl2	178.11 (3)	Cl2—Bi1—Cl4	94.57 (3)
Cl7^i^—Bi1—Cl2	89.16 (3)	Cl3—Bi1—Cl4	94.82 (3)
Cl5^ii^—Bi1—Cl2	91.37 (3)	Bi1^iii^—Cl3—Bi1	98.91 (3)
Cl6^i^—Bi1—Cl3	91.48 (3)	Bi1^iv^—Cl5—Bi1^v^	99.91 (3)
Cl7^i^—Bi1—Cl3	171.71 (3)	H13—O11—H14	110 (6)
Cl5^ii^—Bi1—Cl3	80.58 (2)	H8—O12—H15	103 (7)
Cl2—Bi1—Cl3	88.16 (3)		

Symmetry codes: (i) *x*, *y*, *z*+1; (ii) *x*, −*y*−1/2, *z*+1; (iii) *x*, −*y*−1/2, *z*; (iv) *x*, *y*, *z*−1; (v) *x*, −*y*−1/2, *z*−1.

### Hydrogen-bond geometry (Å, °)

**Table:**

*D*—H···*A*	*D*—H	H···*A*	*D*···*A*	*D*—H···*A*
O11—H13···Cl7^vi^	0.92 (7)	2.32 (7)	3.234 (4)	169 (7)
O11—H14···Cl4^vii^	0.78 (7)	2.56 (8)	3.273 (3)	150 (9)
O12—H8···Cl7^viii^	0.79 (6)	2.78 (7)	3.497 (4)	152 (7)
O12—H15···Cl2^ix^	0.81 (17)	2.81 (8)	3.514 (3)	145 (9)

Symmetry codes: (vi) *x*−1/2, −*y*−1/2, −*z*+1/2; (vii) *x*+1/2, −*y*−1/2, −*z*+3/2; (viii) *x*−1/2, *y*, −*z*+1/2; (ix) *x*−1/2, *y*, −*z*+3/2.
